# P01-043 – Comparative characteristic of FMF and FMF with HSP

**DOI:** 10.1186/1546-0096-11-S1-A46

**Published:** 2013-11-08

**Authors:** H Sargsyan, P Ghazaryan

**Affiliations:** 1 Yerevan State Medical University, Yerevan, Armenia; 2 Haematological Centre, Yerevan, Armenia

## Introduction

According the literature data about Familial Mediterranean Fever (FMF), with the combination of Henoch- Sheilen purpura (HSP), as well as the results of the work done by the former of our studies we attempt to identify and compare membranes aspects of pathogeneses of FMF and combination of FMF with HSP.

## Objectives

The aim of our study is to identify speed of lipid peroxidation (LPO) and the role of degradation of membrane phospholipids (PL) of FMF and combination of FMF with HSP.

## Methods

Clinical studies conducted in 61 non complicated of amyloidosis FMF children in the Republican FMF Children Center, Center “Arabkir”. The age of the patients varies from 5-15. Three patients of FMF are accompanied with HSP. We are selected as a control group of 11 healthy people in practice.Biochemical studies carried out in Hematological Center of Armenia. In erythrocytes of membrane we determine the following index: phospholipase A_2_ activity, intensity of LPO, as well as the level of citotoxic - Lysophatidylcholines( LysoPCH ). The activity of phospholipase A_2_ and LPO was determined by the spektrofotometrik methods. The fractions of separate PL in erythrocyte membranes were carried out by thin-layer chromatography methods.

## Results

The results of our research showed that FMF is followed by rising the activity of phospholipase A_2_ (approximately two times, P<0,001), with sharp increase of citotoxic- LysoPCH (P<0,001) and intensity of LPO (about 3 times, P <0,001).It is noteworthy, that in the second group of our patients (FMF with HSP) these indices will be raised. Therefore, the mentioned follows that the second group of patients treatment is more difficult and requires a special, single-minded approach.

## Conclusion

The results in our experience are discussed to find possible ways of increasing efficiency of treatment FMF with HSP.

## Disclosure of interest

None declared.

